# Supplementary material for: Bacterial IAA-Delivery into Medicago Root Nodules Triggers a Balanced Stimulation of C and N Metabolism Leading to a Biomass Increase
Source: Microorganisms. 2019 Sep 29;7(10):403. doi: 10.3390/microorganisms7100403 (PMC6843515; doi:10.3390/microorganisms7100403)
Supplement: Supplementary file 1 [file microorganisms-07-00403-s001.pdf]

Bacterial IAA-delivery into Medicago Root Nodules Triggers a Balanced Stimulation of C and N Metabolism Leading to Biomass Increase

R. Defez, A. Andreozzi, S. Romano, G. Pocsfalvi, I. Fiume, R. Esposito, C. Angelini, C. Bianco

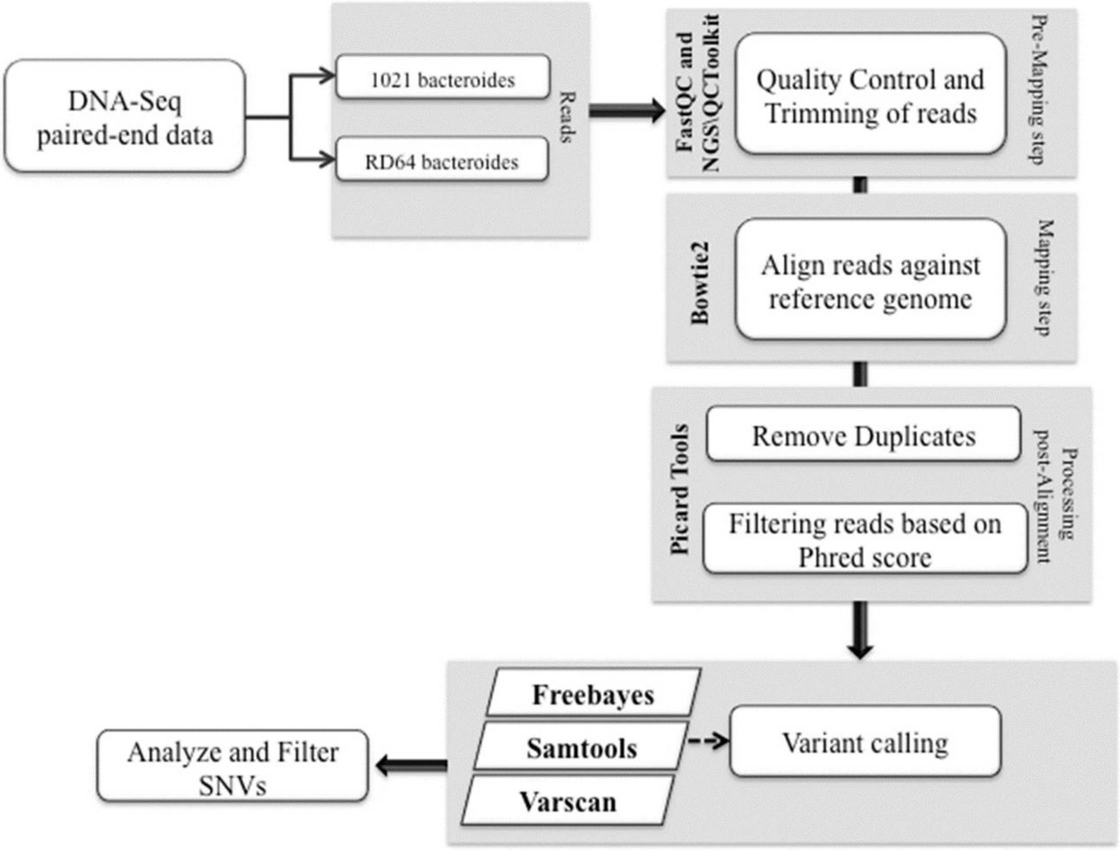

Figure S1. Workflow for the sequencing of *Ensifer melitoti* 1021 and RD64 bacteroids. The bacteroids of both strains were sequenced by a HiSeq2000 Illumina platform, and the resulting short sequencing reads mapped onto the reference genome downloaded from NCBI ((ftp://ftp.ncbi.nlm.nih.gov/genomes/Bacteria/Sinorhizobium_meliloti_1021_uid57603/). The variations between each sample and the reference genome were then identified. Randomly selected mutations were experimentally validated by PCR analysis.

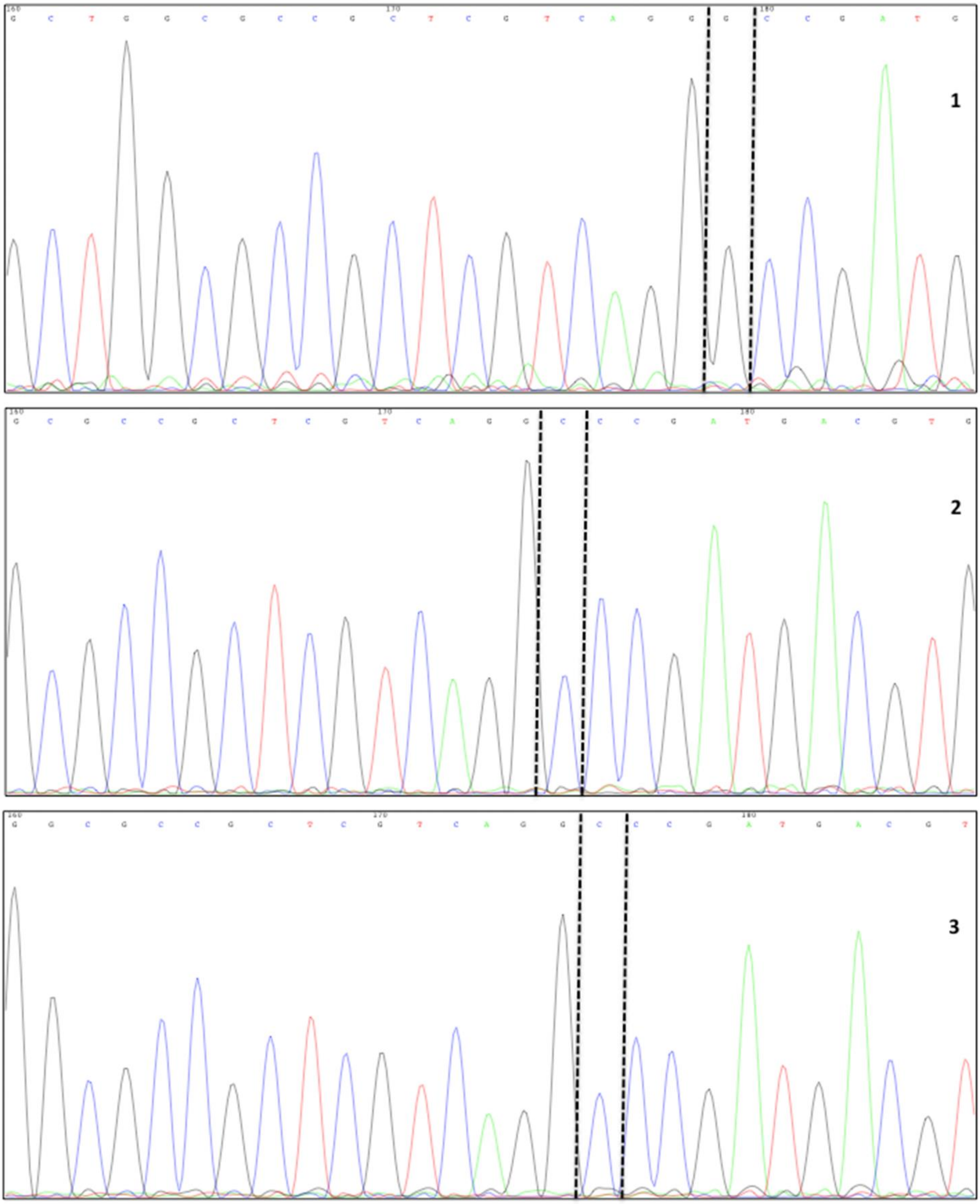

Figure S2. Electropherograms of PCR products obtained for one representative mutation selected for *Ensifer melitoti* 1021 bacteroids. The chromatograms were obtained from three biological replicates (1-3) and the dominant peaks in the sequencing traces which indicate the mutation located in the Sma0233 gene are highlighted with two dashed lines.

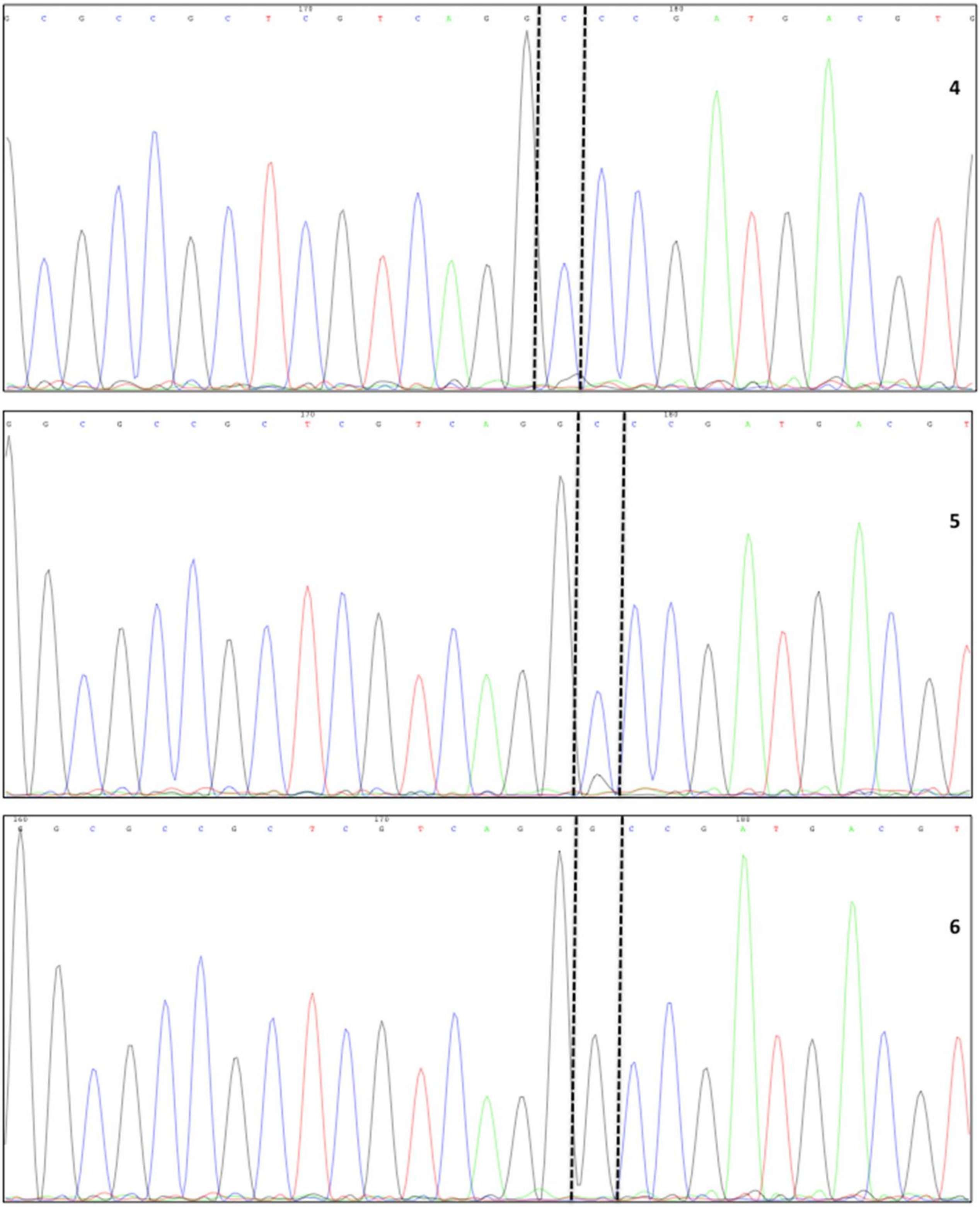

Figure S3. Electropherograms of PCR products obtained for one representative mutation selected for *Ensifer melitoti* RD64 bacteroids. The chromatograms were obtained from three biological replicates (4-6) and the dominant peaks in the sequencing traces which indicate the mutation located in the Sma0233 gene are highlighted with two dashed lines.

Table S1. Overview of the parameters used for single nucleotide variants (SNVs) and small insertion and deletion (INDEL) calling.

Tools	Parameters
Bowtie2	-p 12 --no-discordant –N 0
VarScan	--min-coverage 30 --min-reads2 15 --min-avg-qual 25 --p-value 0.01 --min-var-freq 0.03 --output-vcf
Freebayes	--min-coverage 30 -P 0.99 -C 15 -p 1 -q 25
Samtools	varFilter -Q25
NGSQCToolkit	-q 20 -n 25

Table S2. Primers desigend using Primer3 software (http://primer3.sourceforge.net/) and used in PCR amplifications for Illumina sequence confirmation.

Gene ID	Forward (5′→3′)	Reverse (5′→3′)
SMa0233	AGTTGCACCAGGGCAATATC	CCAGGGAATGTGGAGAAAGA
SMa0461	GCAAGAAGCAGACAGTGGTG	GGCTTATGCTCTTCGATTGC
SMa0673	GACTGGCACATCGAAGATGA	GTACGAAGCCCTCGTCTCTG
SMa0745	CCAAACCGATATGGCGTAAT	GCTTTTCCTTAGCGGTGTCC
SMa0785	CGGTGAGGCTTTTACCTTGA	ATACAGGTAGCGGTGGTTGG
SMa1315	AAGCATCTCCGCGACAAG	ATGACGCGATCCGAGTAGAT
SMa2061	GCAAGAAGCAGACAGTGGTG	GGCTTATGCTCTTCGATTGC
SMb20625	AGATACAGCGACCACTCTGGA	CAAGGAGCATGGCGAGAT
SMb20820	AAGGCGTGGAGATCATCTGT	ACTGATCCGACCAGAACCAC
SMb21085	CCGTCGAGAAAAAGATCGAA	GGCATATGTTCGGAATGCTT
SMb21229	CGACGGGAACAACTATCTGG	ATGACGAGGAAATCGACACC
SMb21356	TTTTCCGAAATAGGCGAAGA	AACCACAGGGAGATCGACAG
SMc00912	TCGGAGCCTATCCTTGACTC	GAACAACCTTGCCGCTTTC
SMc01138	GGCCTGACGAAGTCCTACC	TCACCAAGCAGATCGTTGAG
SMc01312	GTGTGGCGAACTCATGGAC	CAGCAGACGTGATCGTGATG
SMc02823	CGATACGATCACGCTTCTGA	ATGCATAGCGGAAGACCATT

Primer pairs flanking each SNV were designed using Primer3 software (http://primer3.sourceforge.net/) and used for PCR amplification. PCR products were then subjected to Sanger sequencing.

Table S3. Primers used in qRT-PCR analysis.

Gene ID	Gene	Forward (5′→3′)	Reverse (5′→3′)
SMa1227	***fixJ***	CTCGTGACGGACCTGAGAAT	GCAGCAACCAGATGTTCAGA
SMa0815	***nifA***	CCTTGCAAGAGCATTCCTTC	TCTTTGACCTGGCGAGAGTT
SMa0825	***nifH***	TCCACGACCTCCCAAAATAC	CGCACTTGATGCCTCTGTAA
SMa1225	***fixK1***	CATTCTTTCTTTGCCGAAGC	CGCAAAGATCGACGAGAAAT
SMa0762	***fixK2***	AAGCCAAACCACAGTCCATC	CATCTGAAAGGAGGCGGTAG
SMa1220	***fixN1***	AATGGTACGTCGACCTCTGG	AGATTGTTGACGACGTGCAG
SMa1216	***fixO1***	ATCGAGAAGGTCGAGGGAAT	AAAGGGATGGTCGTACATGG
SMa1214	***fixQ1***	CTCGCAATGACGTTGTTCTT	CCTCCTTTAACGGGATGACA
SMa1213	***fixP1***	CAGCTTCTATGCCACGATCA	CGGATTTGCGATGATCTCTT
SMa0765	***fixN2***	GGCATGGTCTTCTCCATCAT	TGTAGTGGCTCAGCGAATTG
SMa0822	***fixA***	TGGCGAAGATTCTCTCCATT	ACTCCACTTGACGACCTTGG
SMa0834	***fdxB***	ATCCAGATGGATGCCAAAAT	ATTGAGCTCACCACCGAAAT
SMc00768	***gltA***	AAGGCCAAAGACAAGAACGA	CGATGTTCGGGTAGAGCTTC
SMc02581	***acnA***	GATTGCCGACTTCAACCAGT	AACGCCCTCCTCCTTGTACT
SMc01124	***icd***	AACCTGGACGAATCGATCAC	TTCCTCGTCGAACACCTTCT
SMa0811	***sucA***	AAGACCGTCGTCCAGCTCTA	CGACCTCCTTCAACTGCTTC
SMc01183	***sucD***	TGTTCCAGACGACCAATGAA	CCTCGTCTTTCAGGAACTGC
SMc00768	***aceA***	GGACGCTATTCCATCTGGTC	CGAGAACGTTGCGATTGTAG
SMc02581	***glcB***	TTCTTTTCCGCCTTCTCTGA	CGATCTCGGAATCGACATTT
SMc00646	***rpoH1***	GTGAGGAAGAGGTCGTCTCG	TCAGAACCTTCATGGCATTG
SMc01235	***uvrA***	CTTCCAGCGCGTAAAGGTAG	GCGAGCGTAAGACAGGTTTC
SMc04231	***uvrB***	CGGTGAAGATCTACGCCAAT	CGAGCATCTCGATGTCGTAA
TC106906	***GH3***	ACTTAACGGTGCCACTGACC	AACTGACGACGGTCCATTTC

Table S4. Conditions for the collision induced dissociation (CID) ion transmissions of the single amino acids determined by using the Thermo Scientific AutoSRM application run under Chromeleon 7.2 software package.

Aminoacid	Time (min)	Precursor ion *m/z*	Product ion *m/z*	Collision energy (eV)	Scan time (sec)
Ala	10	116.1	45	25	0.15
Ala	10	147.1	73.1	15	0.15
Val	13.4	144.1	73	10	0.15
Val	13.4	218.1	73.1	15	0.15
Pro	15.4	142.1	45	30	0.15
Pro	15.4	216.1	147.1	10	0.15
Nor	16.1	158.1	73.1	15	0.15
Nor	16.1	232.1	147.1	10	0.15
Ser	17.2	204.1	188.1	5	0.15
Ser	17.2	204.1	73.1	15	0.15
Thr	17.8	218.1	73.1	15	0.15
Thr	17.8	291.1	101.1	10	0.15
Asp	20.7	232.1	188.1	5	0.15
Asp	20.7	218.1	73.1	15	0.15
Glu	22.5	246.1	128.1	10	0.15
Glu	22.5	230.1	147.1	10	0.15

The conditions reported in the table were determined by using the Thermo Scientific AutoSRM application run under Chromeleon 7.2 software package.

Table S5. Summary of mutations detected as unique to the 1021 bacteroids.

Gene ID[a]	Position	Type	bp Insertion/Deletion	Codon change[b]	Aminoacid change
SMa0056	pSymA:28572-28573	frameshift	A/AG	-	-
SMa0056	pSymA:28580-28581	frameshift	T/TG	-	-
SMa0056	pSymA: 28572-28573	frameshift	G	-	-
SMa0171	pSymA:93633	frameshift	CG/G	-	-
SMa0461	pSymA:245439	missense	-	tcN/tcG	-
SMa0461	pSymA:245660	missense	-	gTt/gAt	V/D
SMa0673	pSymA:359192	missense	-	gGt/gAt	G/D
SMa1132	pSymA:618529	frameshift	GC/C	-	-
SMa1473	pSymA:810470-810472	frameshift	C/CGT	-	-
SMa1608	pSymA:897582	missense	-	cTg/cGg	L/R
SMa2043	pSymA:1156230-1156232	framshift	CGGG/CGG	-	-
SMa2043	pSymA:1156437	framshift	CG/G	-	-
SMa2193	pSymA:1228869	framshift	TA/A	-	-
SMa2307	pSymA:1287071	framshift	CA/A	-	-
SMa2307	pSymA:1287083	framshift	CA/A	-	-
SMa2307	pSymA:1286991-1286992	frameshift	T/CATC	-	-
SMa5034	pSymA:1339737	framshift	AC/C	-	-
SMa5034	pSymA:1339763	framshift	CA/A	-	-
SMb20332	pSymB:335974	frameshift	GT/T	-	-
SMb20332	pSymB:335974	frameshift	-	-	-
SMb20558	pSymB:583800-583801	frameshift	A/AG	-	-
SMb20568	pSymB:1581711-1581712	frameshift	C/CG	-	-
SMb20596	pSymB:1609669-1609670	Frameshift	A/AC	-	-
SMb20625	pSymB:1646596	frameshift	AC/C	-	-
SMb20625	pSymB:1646600-1646601	frameshift	C/CG	-	-
SMb20654	pSymB:1455987-1455988	frameshift	G/GC	-	-
SMb20664	pSymB:1465448-1465449	frameshift	G/GC	-	-
SMb20688	pSymB:1491989-1491990	frameshift	G/GC	-	-
SMb20688	pSymB:1492005	frameshift	GA/A	-	-
SMb20816	pSymB:604526-604527	frameshift	C/CT	-	-
SMb20820	pSymB:609534	missense	-	Cat/Aat	H/N
SMb20841	pSymB:632007-632008	frameshift	G/GC	-	-
SMb20857	pSymB:1262639-1262640	frameshift	A/AG	-	-
SMb20857	pSymB:1262639-1262640	frameshift	G	-	-
SMb21022	pSymB:646477-646478	frameshift	G/GC	-	-
SMb21055	pSymB:688822-688823	Frameshift	G/GC	-	-
SMb21058	pSymB:692914-692915	Frameshift	A/AC	-	-
SMb21063	pSymB:698625-698626	Frameshift	A/AC	-	-
SMb21089	pSymB:732199-732200	frameshift	G/GC	-	-
SMb21089	pSymB: 732199-732200	frameshift	C	-	-
SMb21098	pSymB:741275-741276	Frameshift	A/AG	-	-
SMb21225	pSymB:789801-789802	missense	-	cCG/cGC	P/R
SMb21243	pSymB:811242-811243	missense	-	caCGtt/caGCtt	HV/QL
SMb21257	pSymB:829338-829339	frameshift	A/AG	-	-
SMb21432	pSymB:1362283-1362284	frameshift	A/AG	-	-
SMb21463	pSymB:1390093-1390094	frameshift	G/GC	-	-
SMb21490	pSymB:1413156	frameshift	GC/C	-	-
SMb21496	pSymB:1417679	frameshift	CG/G	-	-
SMb21503	pSymB:1428080-1428081	frameshift	G/GC	-	-
SMb21541	pSymB:1071681-1071682	frameshift	G/GC	-	-
SMb21541	pSymB:1071681	missense	V/L	Gtg/Ctg	
SMb21707	pSymB:1614944	frameshift	AT/T	-	-
SMb21707	pSymB:1614982-1614983	frameshift	G/GC	-	-
SMc00177	Chr:1986166	missense	-	cAg/cCg	Q/P
SMc00858	Chr:929558	missense	-	gCg/gGg	A/G

SMc01312	Chr:1477181	missense	-	gCg/gAg	A/E
SMc02349	Chr:2771499	missense	-	Tgc/Ggc	C/G
SMc02757	Chr:43233	missense	-	Gcg/Ccg	A/P
SMc02793	Chr:799	missense	-	Tcg/Ccg	S/P
SMc02794	Chr:3654130-3654135	frameshift	GTCGCAT/GTCGCA TCGCAT	-	-
SMc02983	Chr:3104144	missense	-	gCg/gGg	A/G
SMc03989	Chr:2987197-2987198	frameshift	G/GC	-	-
SMc04028	Chr:3029642	missense	-	cGc/cCc	R/P
SMc04272	Chr:2202002	missense	-	gCg/gGg	A/G
SMc04357	Chr:2237190-2237193	missense	-	gGTGGc/gACA Ac	GG/DN
SMc04357	Chr:2237194-2237195	missense	-	ggCGgc/ggCAA CGgc	GG/GNG

[a]Genes highlighted in grey were selected for PCR and Sanger sequence analyses. [b]Upper case letters represent mutated bases.

Table S6. Summary of mutations detected as unique to the RD64 bacteroids.

Gene ID§	Position	Type	bp Insertion/Deletion	Codon change¶	Aminoacidchange
SMa0233	pSymA: 130316	missense		Ccc/Gcc	P/A
SMa0745	pSymA:401010	missense		Gtc/Atc	V/I
SMa0785	pSymA:428086	missense		Ggc/Tgc	G/C
SMa0937	pSymA:520887-520890	frameshift		A/AAAAA	
SMa1315	pSymA:718902	missense		Cgc/Ggc	R/G
SMa1539	pSymA: 850708	missense		gCg/gGg	A/G
SMa2061	pSymA: 1162842	missense		Acc/Ccc	T/P
SMb20683	pSymB: 1485681	missense		Gcg/Ccg	A/P
SMb21085	pSymB:723806	missense		gTc/gCc	V/A
SMb21085	pSymB:724102-724103	missense		gtc/gATCAGTtc	V/DQF
SMb21229	pSymB: 793473	missense		gGc/gTc	G/V
SMb21300	pSymB: 881781	missense		cGc/cCc	R/P
SMb21356	pSymB:1022521	missense		aaG/aaT	K/N
SMb21476	pSymB:1400872	frameshift	CC/CCATCCAGC	-	-
SMb21506	pSymB: 1432384	missense		Gcg/Tcg	A/S
SMb21671	pSymB: 1070087-1070088	frameshift	CTTGTGGG	-	-
SMc00722	Chr:2860009	missense		gaG/gaC	E/D
SMc00912	Chr:871102	missense		Gtc/Atc	V/I
SMc01053	Chr:1583930	missense		gTc/gGc	V/G
SMc01138	Chr:413477	missense		cGc/cAc	R/H
SMc01394	Chr: 458847	missense		Gat/Tat	D/Y
SMc01468	Chr: 2315665	missense		tGt/tTt	C/F
SMc01554	Chr:2495818-2495819	missense		gtc/gCAAtc	V/AI
SMc02300	Chr: 673665	missense		Ggc/Tgc	G/C
SMc02303	Chr:676836	missense		cCc/cTc	P/L
SMc02823	Chr:170764	missense		Cgc/Agc	R/S
SMc03257	Chr:3373110	missense		cGc/cTc	R/L

§ Genes highlighted in grey were selected for PCR and Sanger sequence analyses. ¶Upper case letters represent mutated bases.
